# Solubility *vs* Dissolution in Physiological Bicarbonate Buffer

**DOI:** 10.1007/s11095-024-03702-5

**Published:** 2024-05-02

**Authors:** Felix Claussen, Jozef Al-Gousous, Niloufar Salehi, Mauricio A. Garcia, Gordon L. Amidon, Peter Langguth

**Affiliations:** 1 Department of Biopharmaceutics and Pharmaceutical Technology, Johannes Gutenberg University Mainz, 55099 Mainz, Germany; 2 Department of Pharmaceutical Sciences, University of Michigan, 428 Church Street, Ann Arbor, MI 48109 USA; 3 Synthetic Molecule Design & Development, Lilly Research Laboratories, Lilly Corporate Center, Eli Lilly and Company, Indianapolis, IN 46285 USA; 4 Departamento de Farmacia, Escuela de Química y Farmacia, Facultad de Química y de Farmacia, Pontificia Universidad Católica de Chile, 7820436 Santiago, Chile

**Keywords:** bicarbonate buffer, dissolution, ibuprofen, mass transport modelling, phosphate buffer

## Abstract

**Background:**

Phosphate buffer is often used as a replacement for the physiological bicarbonate buffer in pharmaceutical dissolution testing, although there are some discrepancies in their properties making it complicated to extrapolate dissolution results in phosphate to the *in vivo* situation. This study aims to characterize these discrepancies regarding solubility and dissolution behavior of ionizable compounds.

**Methods:**

The dissolution of an ibuprofen powder with a known particle size distribution was simulated *in silico* and verified experimentally *in vitro* at two different doses and in two different buffers (5 mM pH 6.8 bicarbonate and phosphate).

**Results:**

The results showed that there is a solubility *vs*. dissolution mismatch in the two buffers. This was accurately predicted by the in-house simulations based on the reversible non-equilibrium (RNE) and the Mooney models.

**Conclusions:**

The results can be explained by the existence of a relatively large gap between the initial surface pH of the drug and the bulk pH at saturation in bicarbonate but not in phosphate, which is caused by not all the interfacial reactions reaching equilibrium in bicarbonate prior to bulk saturation. This means that slurry pH measurements, while providing surface pH estimates for buffers like phosphate, are poor indicators of surface pH in the intestinal bicarbonate buffer. In addition, it showcases the importance of accounting for the H_2_CO_3_**-**CO_2_ interconversion kinetics to achieve good predictions of intestinal drug dissolution.

**Supplementary Information:**

The online version contains supplementary material available at 10.1007/s11095-024-03702-5.

## Introduction

Being a precondition of active pharmaceutical ingredient (API) absorption, dissolution is often a critical process in drug product performance. This is particularly true when dealing with poorly soluble Biopharmaceutics Classification System (BCS) class II and IV compounds. Therefore, much research has been invested into understanding and modelling the process. Since 1904, the Nernst-Brunner model (which actually is a refinement of the Noyes-Whitney model) has been cornerstone in dissolution modelling. This model can be described by the following equation [1]:

1 $$\frac{d{q}_{diss}}{dt}=\frac{DA}{h} \left({C}_{s}-{C}_{b}\right)=\frac{d{q}_{diss}}{dt}=\frac{DA}{h} \left({C}_{s}-\frac{{q}_{diss}}{{V}_{diss}}\right)$$

q_diss_: is the quantity dissolved at time t
D: is the diffusion coefficient of the solute
A: is the total surface area of the dissolving solute at time t
h: is the thickness of the diffusion layer
C_s_: is the solute concentration at the solid surface
V_diss_: is the volume of the dissolution medium

It has been typically assumed that the surface concentration value equals the saturation solubility, with the net “jumping” of solute molecules from the solid to the solution phase being much faster than the subsequent diffusion, though sometimes this might not be the case [2]. For dissolution in reactive media this assumption has been made too together with another assumption that the typical reaction times are much shorter than the diffusion times.

The reactions most commonly encountered during the dissolution of API’s are the proton-transfer reactions undergone by ionizable molecules in buffered and non-buffered aqueous media. Modelling this dissolution scenario started with the work of Higuchi *et al*. in 1958 [3] and reached a milestone with seminal papers of Mooney *et al*. in 1981 [4, 5]. Mooney *et al*. used the mass balances of the reactions (and implicitly the charge balance) to calculate the surface pH and accordingly the surface concentrations of the different reacting species. The resulting model gives surface API concentrations deviating a bit from the saturation solubility values due to the reacting species having non-equal diffusion coefficients. Another version of this model, using spherical polar co-ordinates, was developed by Ozturk *et al*. in 1988 [6] and combined with the physiologically-based approach of GastroPlus^®^ (which by itself has not included surface pH calculations unlike softwares like SymCyp^®^ and gCOAS^®^) [7]. Nevertheless, the use of saturated slurry concentration as a surface concentration value remained widespread in the pharmaceutical field.

One challenge to those models assuming all species to be at equilibrium came with the bicarbonate buffer, which happens to be the physiological buffer in the human intestine. In the case of this buffer, the very rapid proton transfer reactions are not the only ones involved as shown below:

$${{\varvec{C}}{\varvec{O}}}_{2\boldsymbol{ }({\varvec{a}}{\varvec{q}})}+{{\varvec{H}}}_{2}{{\varvec{O}}}_{({\varvec{l}})}\rightleftharpoons {{\varvec{H}}}_{2}{{\varvec{C}}{\varvec{O}}}_{3\boldsymbol{ }\,({\varvec{a}}{\varvec{q}})}\rightleftharpoons {{{\varvec{H}}}^{+}}_{({\varvec{a}}{\varvec{q}})}+{{\varvec{H}}{\varvec{C}}{\varvec{O}}}_{3({\varvec{a}}{\varvec{q}})}^{-}$$

The interconversion kinetics of carbon dioxide and carbonic acid are not much faster than typical diffusional kinetics with hydration and dehydration times bracketing the diffusional ones [8]. Al-Gousous *et al*. accounted for that in their reversible non-equilibrium (RNE) model by balancing the reaction and diffusion rates ultimately deriving an expression for an apparent interfacial effective pKa value lower than that in the bulk and dependent on the diffusion layer thickness [8]. It was found that “lower effective pKa” at the solute–solvent interface leads to weaker interfacial buffering (especially given that the *in vivo* intestinal bicarbonate molarities are not high) [9] and accordingly considerably slower dissolution rates than in typical pharmaceutical buffers based on phosphate [10]. However, at the same time, the buffering capacity of bicarbonate in bulk was found to be strong (with the bulk pKa of 6.1 being within the intestinal pH range) and even additionally enhanced by its phase-heterogenous nature [11].

A buffer with two different buffer capacities (one in bulk and one at the solid–liquid interface) can have implications for the solubility *vs* dissolution behavior of an ionizable compound. This is because saturation necessarily means equilibrium between all the involved species throughout the system while dissolution does not. Therefore, this work aims to investigate the effect of the aforementioned discrepancies on ionizable API dissolution and solubility in bicarbonate buffer and the associated implications in pharmaceutical development.

## Materials and Methods

### Materials

Ibuprofen 50 powder was received as a gift from BASF SE (Ludwigshafen, Germany). Sodium chloride (Ph. Eur.), Sodium hydrogen carbonate (Ph. Eur.), Sodium dihydrogen phosphate dihydrate (Ph. Eur.) and di-Sodium hydrogen phosphate dodecahydrate (Ph. Eur.) were purchased from Carl Roth GmbH + Co. KG (Karlsruhe, Germany). Sodium dodecyl sulfate (SDS) was obtained from Merck KGaA (Darmstadt, Germany).

### Preparation of Ibuprofen Powder and Determination of the Particle Size Distribution

The ibuprofen 50 powder was sieved dry for ten minutes with a sieve shaker (AS 200 control, Retsch GmbH, Haan, Germany) to narrow down the particle size distribution. The fraction between the sieves with a mesh size of 80 µm and 125 µm respectively was used further on. The particle size distribution analysis was performed with dry powder using a laser diffraction particle size analyzer (Beckman Coulter LS 23 320, Beckman Coulter Inc., Miami, FL, USA). The airflow was set to 420 L/min, the pressure to 740 torr and the obscuration to 6%.

### Microscopy

The microscopic image was taken using an Axioscope 5 microscope with an Axiocam 208 color camera and a N-Achroplan 5x/0,15 objective. All of this was manufactured by Carl Zeiss Microscopy Deutschland GmbH, Oberkochen, Germany and purchased from OptoSys GmbH, Darmstadt, Germany.

### Buffer Preparation

To prepare the dissolution media deionized water was deaerated using a PT-DDS 4 (PharmaTest, Hainburg, Germany) at 30°C for 2 h. The water was allowed to cool down overnight. After dissolving the buffer salts in deaerated water at room temperature, the pH of the 5 mM phosphate buffer was adjusted to 6.8 ± 0.05 using 0.1 N HCl/NaOH and a WTW pH 538 pH meter with an WTW SenTix^®^81 plus electrode (both Xylem Inc., Washington, D. C., USA) as described by Blechar *et al*. [12]. The ionic strength of both buffers was adjusted to 154 mM with NaCl. The pH values were also checked after reaching a temperature of 37°C. The pH of the 5 mM bicarbonate buffer (molarity selected based on average of duodenal bicarbonate molarity in two publications [13, 14]) was adjusted with a mixture of CO_2_ and air in the dissolution apparatus, as described by Al-Gousous *et al.* [15].

### Dissolution Test

The dissolution test was performed in triplicate using a PTW S III dissolution tester (PharmaTest, Hainburg,Germany). The dissolution of the ibuprofen powder was tested in a paddle apparatus (USP Type II apparatus) at 50 rpm. The test was performed in a total volume of 900 mL at 37°C. The ibuprofen was pre-dispersed in a tube by fractionized addition of 10 mL deionized water containing sodium dodecyl sulfate (SDS) at a concentration equal to 25% of the critical micelle concentration (CMC) [16] and under continuous stirring. The suspension was added to the 880 mL of buffer already in the dissolution vessel. Afterwards the tube was rinsed with 10 mL of buffer, which was also added to the dissolution vessel. At predefined time points, 5 mL samples were withdrawn with a syringe and not replaced with medium. The samples were filtered through 0.45 µm PTFE filters (purchased from Carl Roth GmbH, Karlsruhe, Germany) and quantified spectrophotometrically (UV–visible spectrophotometer, Thermo Scientific EVOLUTION 201, ThermoFisher Scientific, Waltham, MA, USA) at the wavelength of 222 nm. During the experiment with bicarbonate buffer, the dissolution media was sparged with a mixture of CO_2_ (SAPHIR, Air Liquide S.A., Paris, France) and air to adjust the pH accordingly, and the dissolution was started after the pH in all three vessels was stabilized at 6.8 ± 0.05 for at least 15 min [17]. The pH was measured continuously in one of the vessels during dissolution, and, for the 2000 mg dose, the pH was also measured at the end of the dissolution in all three vessels.

### *In Silico* Simulations

For surface pH, the equations of Mooney’s model [4, 5] in phosphate and the RNE model [8] in bicarbonate without deleting the bulk drug concentration term (to account for saturation at high doses) were used. These were combined with the Nernst-Brunner model [1] adapted for particle dissolution. The diffusion layer thickness (or more appropriately resistance to mass transfer) was calculated using the Wang-Flanagan method in a way similar to what was done Avdeef *et al*. [18, 19]. The detailed equations and the used values for physicochemical parameters are shown in the supplementary material. The equations were solved using the NDSolve command in Mathematica version 13.1 (Wolfram Research, Champaign, IL, USA).

## Results

### Particle Size Distribution

**Fig. 1:**
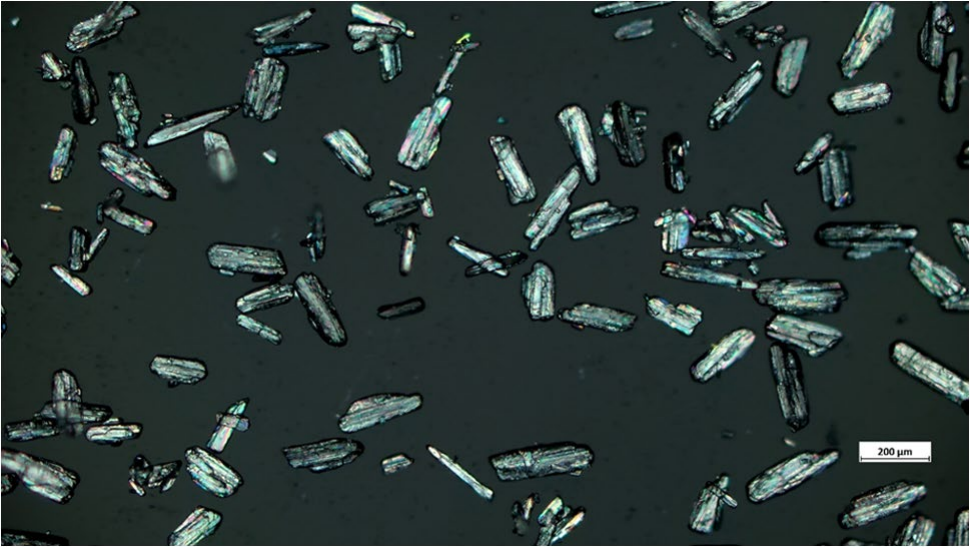
Microscopic image of the ibuprofen particles.

Table I shows the particle size distribution results for the ibuprofen powder. The median particle radius was 34.8 µm with an interquartile range of 21.2 to 50.9 µm. Despite the sieving there were particles above and below the used mesh sizes. This could be due to the need for longer sieving time and the aspect ratio of the particles not being one, while LPD assumes the particles to be spherical (See Fig. 1).

**Table I:** The Particle Size Distribution of the Sieved Ibuprofen Powder. Values are Mean ± Standard Deviation

% Cumulative undersize	Radius (µm)
10	10.8 ± 0.40
25	21.2 ± 0.46
50	34.8 ± 0.49
75	50.9 ± 0.51
90	67.2 ± 0.97
99	94.9 ± 3.55

### Dissolution

**Fig. 2:**
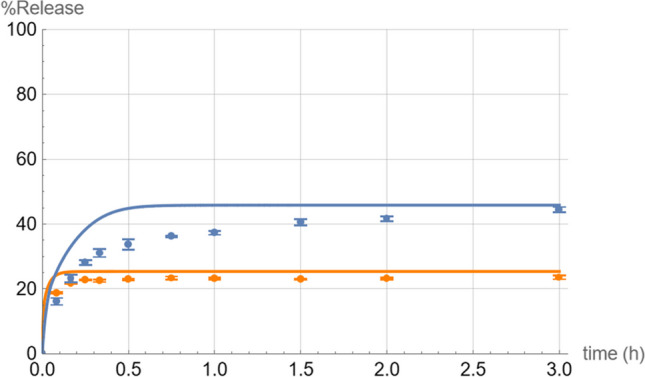
Dissolution profiles of 2000 mg ibuprofen in 5 mM phosphate buffer (orange) and 5 mM bicarbonate buffer (blue) at a starting pH of 6.8. The lines are the theoretical simulated curves while the dots are the experimental data points (*n* = 3).

The experimental results confirmed the Mathematica^®^ simulations’ prediction that ibuprofen has a higher (roughly double) saturation solubility in 5 mM pH 6.8 bicarbonate compared to 5 mM pH 6.8 phosphate (Fig. 2).

**Fig. 3:**
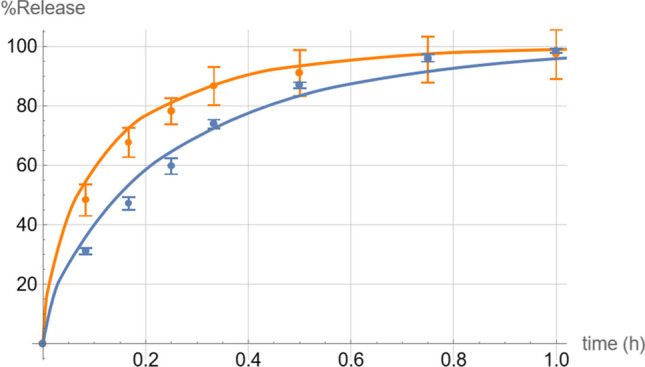
Dissolution profile of 200 mg ibuprofen in 5 mM phosphate buffer (orange) and 5 mM bicarbonate buffer (blue) at a starting pH of 6.8. The solid curves are the theoretical simulations while the dots are the experimental data points (*n* = 3).

The experimental results also confirmed the *in silico* prediction, that in the absence of saturation, dissolution in 5 mM phosphate is faster than in bicarbonate, taking almost half the time to reach 50% dissolution (Fig. 3) despite the lower saturation solubility. This is further supported by the initial dissolution (before saturation effects take their toll) being faster for phosphate also at the 2000 mg dose, as seen at the 5-min point (the difference is statistically significant with the t-test giving a p-value of less than 0.05). The faster initial dissolution in phosphate at the 2000 mg dose was also predicted by our *in silico* model, albeit with an earlier intersection of the two curves. In general, dissolution profile predictions for the 200 mg dose were superior to those for the 2000 mg dose (but the final saturation solubility levels reached by the 2000 mg dose were accurately predicted as seen in Table II).

**Table II:** Values of Major Simulated and Experimental Parameters

		Bicarbonate 5 mM pH 6.8	Phosphate 5 mM pH 6.8
Bulk buffer capacity (mM/ΔpH)		11.52^b^	2.87^a^
CO_2_ partial pressure (atm)		0.042	Not relevant
Initial surface pH^c^,		4.81–5.31	5.50
Initial surface solubility of ibuprofen (mM)^b^		0.98–2.5	3.72
200 mg dose	Final bulk pH, simulated	6.70	6.41
	Final bulk pH, experimental (*n* = 1)	6.69	6.36
2000 mg dose	Final bulk pH, simulated	5.63	5.35
	Final bulk pH, experimental (*n* = 3)	5.67 ± 0.00	5.38 ± 0.00
	Final bulk solubility (mM), simulated	4.93	2.72
	Final bulk solubility (mM), experimental (*n* = 3)	4.79 ± 0.09	2.54 ± 0.06

^a^ Van Slyke calculation method for phosphate [20]

^b^ Al-Gousous *et al*. [11] and James N. Butler [21] calculation method for sparged bicarbonate

^c^ Range for bicarbonate because of particle size dependence

**Fig. 4:**
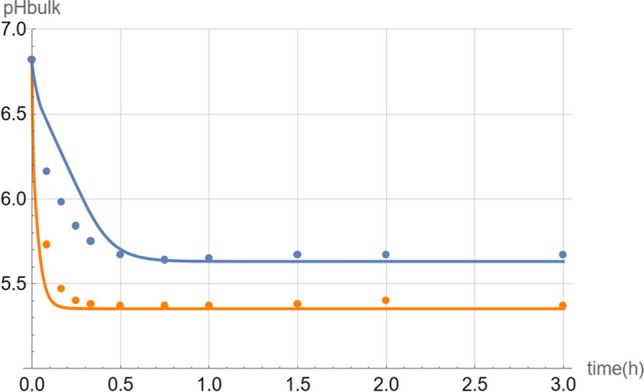
Change of bulk pH during the dissolution of 2000 mg ibuprofen in 5 mM phosphate buffer (orange) and 5 mM bicarbonate buffer (blue) at a starting pH of 6.8. The solid curves are the theoretical simulations while the dots are the experimental data points.

Besides the dissolution of ibuprofen, the Mathematica^®^ simulation also predicted the decrease of the pH curve under non-sink conditions (Fig. 4).

Some values of major simulated and experimental parameters are summarized in Table II.

## Discussion

### Dissolution and Solubility Predictions

The RNE and Mooney model-based simulations coded in Mathematica gave fair to very good predictions of the dissolution and solubility behavior of ibuprofen in bicarbonate and phosphate buffers. The dissolution rate predictions for the 2000 mg dose in bicarbonate were the least accurate. This is because the model did not incorporate a finite mass transfer rate of carbon dioxide between the aqueous and gaseous phases in the sparged system. Instead, it assumed that the equilibration between dissolved and gaseous carbon dioxide would be instantaneous thus underestimating the accumulation kinetics of the carbon dioxide in the bulk (generated by the reaction between ibuprofen and bicarbonate). However, the final solubility was still highly accurately predicted since long enough time was given for equilibration. The maximum relative prediction error for dissolution in bicarbonate for the 2000 mg dose was 57% at the earliest timepoint of 5 min. The issues associated with CO_2_ volatility could be made less problematic by adopting the floating lid method proposed by Sakamoto and Sugano, which would obviate the need for sparging during drug product dissolution [22].

**Fig. 5:**
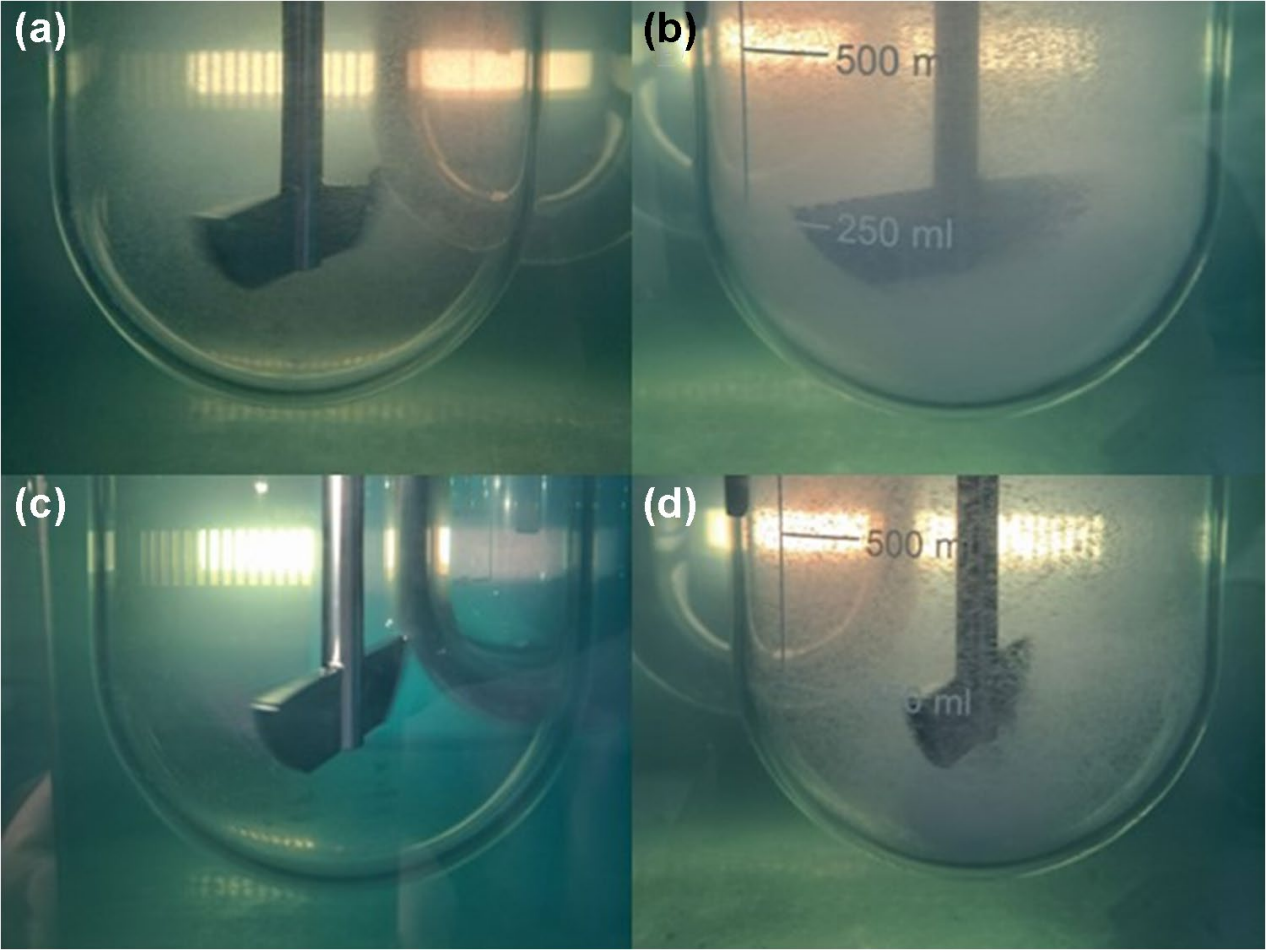
Distribution of the ibuprofen particles during the dissolution experiment. The images show 200 mg (**a**) and 2000 mg (**b**) at t_3min_ and 200 mg (**c**) and 2000 mg (**d**) at t_60min_.

An additional factor contributing to those deviations in bicarbonate (and in this case also phosphate) at the 2000 mg dose, was the formation of a particle-rich cloud (not a real cone or heap but rather a cloud) below the paddle (See Fig. 5d). This was not observed for the 200 mg dose in neither buffer (probably due to the extensive dissolution as well as lower particle “concentration”), which explains why highly superior predictions for the 200 mg dose were observed in both buffers (the maximum relative prediction error for the dissolved fraction of the 200 mg dose was 15.6% and was observed in bicarbonate at the 5 min timepoint).

**Fig. 6:**
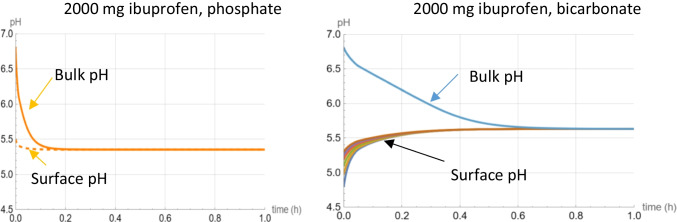
Simulated surface *vs* bulk pH profiles in bicarbonate and phosphate during the dissolution of the 2000 mg dose. Six different surface pH curves exist for bicarbonate representing the surface pH of the six particle size fractions since, in bicarbonate, surface pH varies with particle size [10].

An important finding is related to the reversed rank order of saturation solubility *vs* dissolution kinetics in the absence of saturation in the two buffers. This solubility *vs* dissolution rate mismatch may seem counter-intuitive at the first glance, but it can be easily explained. It is because while the initial surface pH in phosphate is not that far from the pH at saturation, bicarbonate shows a large gap between the initial surface and saturation pH values. The reason behind that lies in the fact that, while in phosphate buffer all the reactions occurring at the interface are at equilibrium, the H_2_CO_3_-CO_2_ interconversion fails to reach equilibrium at the surface of the dissolving ibuprofen particles in bicarbonate prior to saturation. As a result, bicarbonate will act as if it had two different buffer capacities: One at the interface and one in bulk. This results in the initial surface pH being much lower than  the bulk pH at saturation. As saturation is approached, this gap closes as the whole system (both the bulk and, consequently, the interface) approaches equilibrium (Fig. 6).

One *in silico* observation might seem counter-intuitive at first glance, namely the increase in the surface pH of ibuprofen in bicarbonate despite the decreasing bulk pH with drug dissolution and accumulation as shown in Fig. 6. This is because as the drug accumulates in the bulk fluid, the system approaches equilibrium as saturation is being approached and the main buffer role shifts from the consumed bicarbonate to the accumulating drug molecules. And since the equilibrium pH is higher than the initial surface pH, the surface pH will be increasing with time.

### Implications

This has important implications for surface pH estimation methods. Slurry pH has typically been the method of choice [23–25] While this works fine for ordinary buffers like phosphate [25], it leads to erroneous estimates in bicarbonate. Therefore, surface pH in bicarbonate needs to be estimated either theoretically through mathematical modelling or through the use of indicator dyes which would be a complicated procedure. The major limitation of theoretical models like the RNE model used in this work is the possible interfacial self-association of some drugs when the interfacial concentrations exceed a certain threshold. This can be accounted for by characterizing the phase-solubility behavior as a function of pH [26]. Another surface pH estimation method could be through matching intrinsic dissolution rates in bicarbonate to those in phosphate. An additional implication is related to solubility estimation in human intestinal fluid (HIF). This gap between the surface and bulk saturation pH values means that solubilities in HIF should be taken with caution when used to predict dissolution rates. This is because solubility measurements are associated with an equilibrium, which is a poor indicator of interfacial buffering by bicarbonate (the principal buffer of HIF) when saturation is not reached.

Another complication is related to surrogate buffers. The concept of replacing bicarbonate with an appropriately designed surrogate phosphate buffer has been proposed [27, 28] and successfully applied [29] before. This provides a way for circumventing the technical difficulties associated with bicarbonate by designing a phosphate buffer giving a similar surface pH of the drug. This, however, applies rather to BCS class II than BCS class IV compounds. For a BCS class II compound like ibuprofen, the high permeability of the API molecule prevents accumulation in the bulk of intestinal fluid. This means that having the surface pH predicted correctly is sufficient for *in vivo*-predictive *in silico* modelling methods and surrogate *in vitro* testing method design. However, the situation can be different with a BCS class IV compound, especially if the dose is high. In this case, the accumulation of the poorly permeating drug can result in large bulk pH shifts that need to be accounted for during predictive *in silico* modelling and *in vitro* testing method design. Since the discrepancy between the initial surface pH and the saturation pH is large in bicarbonate, designing the surrogate *in vitro* buffer will involve a difficult choice between basing the design on the initial surface pH or the changing bulk pH value near saturation. This could also be affected by the pH-restoring action of the intestinal epithelial ion transporters *in vivo*, which needs to be investigated further.

On a more positive note, mechanistically sound surface pH and accordingly interfacial solubility models for bicarbonate, can help in expanding biowaivers to weakly acidic BCS class II (also called BCS class IIa [30]) compounds, an idea that has been floated before [30, 31]). In this case, interfacial solubility values in bicarbonate can guide us to compounds for which the dissolution rate in intestinal bicarbonate can realistically be so fast that the gastric emptying would be the rate limiting step of the overall absorption if formulation factors allow it. For instance, if the ibuprofen powder tested in this study had had a 60% smaller particle size (this would mean a median particle size of 28 µm which not unrealistic) it would have probably passed the very rapidly dissolving criterion. This shows in a mechanism-based manner that achieving *in vivo* intestinal dissolution rates that make gastric emptying the rate-limiting step of overall absorption is possible for some BCS class II acidic compounds (even at the relatively low bicarbonate molarities present in the duodenum), and thus the idea of biowaiver extension to them warrants consideration. Furthermore, availability of mechanistic models can help to guide the formulators to develop products that fulfill the necessary criteria for such a (for the time being) hypothetical biowaiver.

### Limitations

**Fig. 7:**
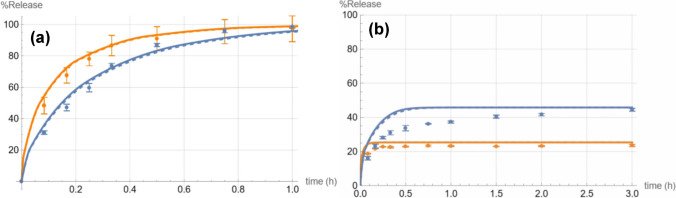
Predicted *vs* observed dissolution of 200 mg ibuprofen (**a**) and 2000 mg ibuprofen (**b**) in 5 mM phosphate buffer (orange) and 5 mM bicarbonate buffer (blue) when assuming almost complete dimerization (dashed curve) of the non-ionized ibuprofen species *vs* no dimerization (solid curves). In phosphate the dashed curve is partially obscured by the solid one at the 200 mg dose and completely at the 2000 mg dose.

Two limitations were discussed previously: The assumption of immediate equilibration between the dissolved and gaseous CO_2_ and the particle-rich cloud formation below the paddle at the 2000 mg dose. Additional limitations include:

- Ibuprofen free acid can dimerize with a log K_dimer_ of 4.67 ± 0.07 at 22°C [32]. This would have, at least in theory, no effect on the pH solubility profile (especially if the pKa were estimated by a solubility method, which was the case for the pKa estimate used here) since the impacts of the errors in intrinsic solubility and pKa estimates for the monomeric form will cancel out each other [33]. However, it would mean that the diffusivity of the free acid dimer would be lower than that of the monomeric free acid and, accordingly, of the ionized species for which the same diffusivity as that of the protonated monomer was assumed. The effect, as shown in the simulation results outlined in Fig. 7, is small because most of the flux under the relevant surface pH conditions pertains to the ionized species. The ionized form does not show evidence of aggregation at surface pH values lower than 6 [26]. In addition, the change in diffusivity is not large since the relationship with molecular weight is not linear [34]. Since the dimerization constant value at 37°C is not available, the simulations were performed assuming the most extreme case of almost all the protonated molecules being dimerized.
- Our model did not account for the possible effects of electrical field gradients generated during ion diffusion. However, with the background electrolyte (NaCl) concentration being at least 29.8 times higher than that of any fluxing ionic species, this effect is expected to be very small [35]. In addition, the differences in the diffusivities between the ionic species whose movement constitutes the dominant portion of ionic fluxes (HCO_3_^−^ and deprotonated ibuprofen in the case of bicarbonate and H_2_PO_4_^−^, HPO_4_^2−^ and deprotonated ibuprofen in the case of phosphate) is not as large as in the case of unbuffered media where the role of the fluxes of H^+^ and OH^−^ fluxes and their high diffusion coefficients would gain in prominence.
- The shape of the particles is not spherical but elongated prismatic. The effect of the different geometries is reduced by the LPD calculating an equivalent spherical radius (though not completely eliminated because the surface area to volume ratio is not exactly the same, which is a limitation of this study). The sphericity assumption will underestimate the surface area to volume ratio, but the impact of this is opposed by that of the underestimation of the resistance to mass transfer because of the introduction of a higher (assumed) curvature. Curvature enhances the flux density at the interface compared to a flatter surface [18]. A detailed analysis of those effects is beyond the scope of this work.

## Conclusion

While fairly accurate for must buffers, saturated slurry pH is a poor indicator of surface pH in bicarbonate and can lead to erroneous conclusions regarding intestinal drug dissolution. Alternatively *in silico* and/or *in vitro* approaches can be used to overcome this problem. Accounting for this issue is needed to improve predictions of intestinal dissolution of ionizable BCS class II compounds and for any attempt to extend biowaivers to BCS class IIa drugs.

### Supplementary Information

Below is the link to the electronic supplementary material.

Supplementary file1 (DOCX 142 kb)

## Data Availability

The datasets generated during and/or analyzed during the current study are available from the corresponding author on reasonable request.
