# Supplementary material for: Solubility vs Dissolution in Physiological Bicarbonate Buffer
Source: Pharm Res. 2024 May 2;41(5):937–45. doi: 10.1007/s11095-024-03702-5 (PMC11116206; doi:10.1007/s11095-024-03702-5)
Supplement: Supplementary file 1 — Supplementary file1 (DOCX 142 kb) [file 11095_2024_3702_MOESM1_ESM.docx]

# Supplemental material: Dissolution model equations

For particle dissolution, Nernst-Brunner model was used with the surface concentration (C_s_) being set to equal the intrinsic solubility (is) plus the interfacial ionized drug concentration which is the intrinsic solubility multiplied by drug Ka and divided by the surface proton molarity ([H^+^]_s_) since the ionized and ionized forms are at equilibrium.

| $\frac{dq_{diss}}{dt}=\frac{DA}{h} \left( C_{s}-C_{b} \right)=\frac{dq_{diss}}{dt}=\frac{DA}{h} \left( C_{s}-\frac{q_{diss}}{V_{diss}} \right)=\frac{DA}{h} \left( is\times(1+\frac{K_{a}^{HA}}{\left[ H^{+} \right]_{s}})-\frac{q_{diss}}{V_{diss}} \right)$ | (S1) |
| --- | --- |

Where q_diss_ is the quantity dissolved at time t, D is the diffusion coefficient of the drug, A is the total surface area of the drug particles.

| $A=N\times4\pi r^{2}$ | (S2) |
| --- | --- |

Where N is the number of dissolving drug particles and r is the particle radius.

| $N=\frac{Dose}{\rho Vparticle.ini}=\frac{3Dose}{4\pi r_{ini}^{3}\rho}$ | (S3) |
| --- | --- |

Where ρ is the density of a drug particle, V_particle.ini_ is the initial particle volume and r_ini_ is the initial particle radius.

| $Vparticle=\frac{4}{3}\pi r^{3}=\frac{q_{undiss}}{\rho N}$ | (S4) |
| --- | --- |

| $r=\sqrt[3]{\frac{3Vparticle}{4\pi}}=\sqrt[3]{\frac{3q_{undiss}}{4\pi\rho N}}$ | (S5) |
| --- | --- |

Where q_undiss_ is the quantity yet to be dissolved.

Substitute (S3) into (S5):

| $r=\sqrt[3]{\frac{q_{undiss}r_{ini}^{3}}{Dose}}$ | (S6) |
| --- | --- |

Substituting (S6) and (S3) into (S2):

| $A=\frac{3Dose}{4\pi r_{ini}^{3}\rho}\times4\pi{(\frac{q_{undiss}r_{ini}^{3}}{Dose})}^{2/3}=\frac{3Dose}{r_{ini}\rho}\times{(\frac{q_{undiss}}{Dose})}^{2/3}$ | (S7) |
| --- | --- |

| $Dose=q_{diss}+q_{undiss}$ | (S8) |
| --- | --- |

| $q_{undiss}=Dose-q_{diss}$ | (S9) |
| --- | --- |

Substitute (S9) into (S7):

| $A=\frac{3Dose}{r_{ini}\rho}\times{(1-\frac{q_{diss}}{Dose})}^{2/3}$ | (S10) |
| --- | --- |

Substitute (S10) into (S1):

| $\frac{dq_{diss}}{dt}=\frac{3 D Dose}{hr_{ini}\rho}{(1-\frac{q_{diss}}{Dose})}^{2/3} \left( is\times(1+\frac{K_{a}^{HA}}{\left[ H^{+} \right]_{s}})-\frac{q_{diss}}{V_{diss}} \right)$ | (S11) |
| --- | --- |

To account for drug particle polydispersity, the dose was divided into six fractions based on d0 (zero by default), d10, d25, d50, d75, d90 and d99 values of the cumulative volume particle size distribution values obtained from laser diffractometry. It was assumed that all particles have identical densities. Each fraction was assigned a diameter equal to the mid-point of the size interval. Accordingly, equation (S11) became:

| $\frac{dq_{diss}}{dt}=\sum_{i=1}^{n=6} (\frac{3 D f_{i} Dose}{h_{i} {r_{ini}}_{i}\rho}\left( 1-\frac{{q_{diss}}_{i}}{f_{i} Dose} \right)^{\frac{2}{3}} \left( is\times(1+\frac{K_{a}^{HA}}{\left[ H^{+} \right]_{s}})-\frac{\sum_{i=1}^{n=6} {q_{diss}}_{i}}{V_{diss}} \right))$ | (S12) |
| --- | --- |

Where $f_{i}$ is the mass fraction of each size fraction, ${r_{ini}}_{i}$ is its initial radius (size interval mid-point), $h_{i}$ is the corresponding diffusion layer thickness and ${q_{diss}}_{i}$ is the quantity dissolved out of the corresponding fraction. In bicarbonate, since the surface pH is dependent on diffusion layer thickness and accordingly on particle size, the surface proton molarity was also assigned an index i.

For calculating the diffusion layer thickness, the Wang-Flanagan model [1] was used. In this regard, it might be better to describe this diffusion layer thickness as a resistance mass transfer term, since it combines the aqueous boundary layer thickness (ABL), which is assumed to remain constant during dissolution (i.e. particle size-independent), with the effect of the particle curvature on the flux density as follows:

| $\frac{1}{h_{i}}=\frac{1}{ABL}+\frac{1}{r_{i}}$ | (S13) |
| --- | --- |

Substituting equation (S6), after adapting it to the particle size fraction of the dose case, into (S13):

| $\frac{1}{h_{i}}=\frac{1}{ABL}+\frac{1}{\sqrt[3]{\frac{{q_{undiss}}_{i} r_{{ini}_{i}}^{3}}{f_{i} Dose}}}$ | (S14) |
| --- | --- |

Substituting equation (S8), after adapting it to the particle size fraction of the dose case, into (S14) and re-arranging gives

| $\frac{1}{h_{i}}$*=*$\frac{1}{ABL}+\frac{1}{\sqrt[3]{r_{{ini}_{i}}^{3}(1-\frac{{q_{diss}}_{i}}{f_{i} Dose})}}$ | (S15) |
| --- | --- |

For estimating ABL thickness, an approach based on the work of Avdeef et al. [2] was adapted. The ABL thickness was simply estimated by assuming the rotating disk at 50 rpm to roughly represent the situation with very large particles (zero curvature and so infinitely large radius of curvature), where the contribution of the of the r term in the ABL equation becomes negligible. For this purpose, the Levich equation was used:

| $ABL=1.612 D^{1/3}\omega^{-1/2}\nu^{1/6}$ | (S16) |
| --- | --- |

Where D is the drug diffusion coefficient, ω is the angular velocity, ν is the kinematic viscosity. The use of Levich equation to estimate ABL introduces and additional limitation since the fluid velocity profiles around a particle in a paddle apparatus are not identical to the rotating disk case. The presence of the *1/r_i_* term in equation (S13) combined with the not so large particle size reduces the impact of this as it means that a large part of the resistance to mass transfer estimate is not directly affected by this limitation but is rather a function of the particle size. In addition, the effect of the spinning of the hypothetical very large particle assumed for calculating ABL might decrease the impact of the difference in hydrodynamics.

For calculating the change of bulk pH with time in phosphate buffer, the first and the third ionizations of phosphoric acid were ignored and the derivation proceeded as follows:

Charge balance in phosphate buffer bulk (ignoring the first and third ionizations of phosphoric acid):

| $\left[ M^{+} \right]+\left[ H^{+} \right]=\left[ {OH}^{-} \right]+\left[ H_{2}{PO}_{4}^{-} \right]+2\left[ {HPO}_{4}^{-} \right]+[A^{-}]$ | (S17) |
| --- | --- |

Since the total buffer concentration is constant:

| $C_{buffer}=\left[ H_{2}{PO}_{4}^{-} \right]+\left[ {HPO}_{4}^{-} \right]$ | (S18) |
| --- | --- |

| $K_{a}^{P}=\frac{\left[ {HPO}_{4}^{-} \right]\left[ H^{+} \right]}{\left[ H_{2}{PO}_{4}^{-} \right]}$ | (S19) |
| --- | --- |

Making the molarity of dihydrogen phosphate ion the subject of the above formula (S19), substituting into (S18) and re-arranging give:

| $\left[ {HPO}_{4}^{-} \right]=\frac{K_{a}^{P} C_{buffer}}{K_{a}^{P}+\left[ H^{+} \right]}$ | (S20) |
| --- | --- |

Substituting (S20) into (S18) and re-arranging gives:

| $\left[ H_{2}{PO}_{4}^{-} \right]=\frac{\left[ H^{+} \right] C_{buffer}}{K_{a}^{P}+\left[ H^{+} \right]}$ | (S21) |
| --- | --- |

Given that:

| $K_{a}^{HA}=\frac{\left[ HA \right]\left[ H^{+} \right]}{\left[ A^{-} \right]}$ | (S22) |
| --- | --- |

Taking into account the mass balance:

| $q_{diss}=\left[ HA \right]+\left[ A^{-} \right]$ | (S23) |
| --- | --- |

| $\left[ A^{-} \right]=\frac{K_{a}^{HA}q_{diss}}{K_{a}^{HA}+\left[ H^{+} \right]}$ | (S24) |
| --- | --- |

Also:

| $\left[ {OH}^{-} \right]=\frac{K_{w}}{\left[ H^{+} \right]}$ | (S25) |
| --- | --- |

Substituting equations (S18), (S20), (S24) and (S25) into (S17) gives:

| $\left[ M^{+} \right]+\left[ H^{+} \right]=\frac{K_{w}}{\left[ H^{+} \right]} +C_{buffer}+\frac{K_{a}^{P} C_{buffer}}{K_{a}^{P}+\left[ H^{+} \right]} +\frac{K_{a}^{HA}q_{diss}}{K_{a}^{HA}+\left[ H^{+} \right]}$ | (S26) |
| --- | --- |

Differentiating with respect to time:

| $\frac{d\left[ H^{+} \right]}{dt}=-\frac{K_{w}}{\left[ H^{+} \right]^{2}} \frac{d\left[ H^{+} \right]}{dt}-\frac{K_{a}^{P} C_{buffer}}{\left( K_{a}^{P}+\left[ H^{+} \right] \right)^{2}} \frac{d\left[ H^{+} \right]}{dt}+\frac{K_{a}^{HA}\left( K_{a}^{HA}+\left[ H^{+} \right] \right)}{\left( K_{a}^{HA}+\left[ H^{+} \right] \right)^{2}}\frac{dq_{diss}}{dt}-\frac{K_{a}^{HA}q_{diss}}{\left( K_{a}^{HA}+\left[ H^{+} \right] \right)^{2}}\frac{d\left[ H^{+} \right]}{dt}$ | (S27) |
| --- | --- |

| $\frac{d\left[ H^{+} \right]}{dt}(1+\frac{K_{w}}{\left[ H^{+} \right]^{2}}+\frac{K_{a}^{P} C_{buffer}}{\left( K_{a}^{P}+\left[ H^{+} \right] \right)^{2}}+\frac{K_{a}^{HA}q_{diss}}{\left( K_{a}^{HA}+\left[ H^{+} \right] \right)^{2}})= \frac{K_{a}^{HA}\left( K_{a}^{HA}+\left[ H^{+} \right] \right)}{\left( K_{a}^{HA}+\left[ H^{+} \right] \right)^{2}}\frac{dq_{diss}}{dt}$ | (S28) |
| --- | --- |

| $\frac{d\left[ H^{+} \right]}{dt}=\frac{K_{a}^{HA}\left( K_{a}^{HA}+\left[ H^{+} \right] \right)}{\left( K_{a}^{HA}+\left[ H^{+} \right] \right)^{2}}\frac{dq_{diss}}{dt}\div(1+\frac{K_{w}}{\left[ H^{+} \right]^{2}}+\frac{K_{a}^{P} C_{buffer}}{\left( K_{a}^{P}+\left[ H^{+} \right] \right)^{2}}+\frac{K_{a}^{HA}q_{diss}}{\left( K_{a}^{HA}+\left[ H^{+} \right] \right)^{2}})$ | (S29) |
| --- | --- |

For bulk pH in bicarbonate the second ionization of carbonic acid was ignored and the derivation proceeded as follows:

| $\left[ M^{+} \right]+\left[ H^{+} \right]=\left[ {OH}^{-} \right]+\left[ {HCO}_{3}^{-} \right]+[A^{-}]$ | (S30) |
| --- | --- |

| $K_{a}^{{CO}_{2}}=\frac{\left[ {HCO}_{3}^{-} \right]\left[ H^{+} \right]}{\left[ {CO}_{2} \right]}$ | (S31) |
| --- | --- |

Assuming that sparging maintains a constant partial pressure of carbon dioxide re-arranging (S31), and substituting it together with (S24) and (S25) into (S30) gives:

| $\left[ M^{+} \right]+\left[ H^{+} \right]=\frac{K_{w}}{\left[ H^{+} \right]} +\frac{\left[ {CO}_{2} \right]K_{a}^{{CO}_{2}}}{\left[ H^{+} \right]}+\frac{K_{a}^{HA}q_{diss}}{K_{a}^{HA}+\left[ H^{+} \right]}$ | (S32) |
| --- | --- |

Differentiating with respect to time and re-arranging:

| $\frac{d\left[ H^{+} \right]}{dt}=\frac{K_{a}^{HA}\left( K_{a}^{HA}+\left[ H^{+} \right] \right)}{\left( K_{a}^{HA}+\left[ H^{+} \right] \right)^{2}}\frac{dq_{diss}}{dt}\div(1+\frac{K_{w}}{\left[ H^{+} \right]^{2}}+\frac{\left[ {CO}_{2} \right]K_{a}^{{CO}_{2}}}{\left[ H^{+} \right]^{2}}+\frac{K_{a}^{HA}q_{diss}}{\left( K_{a}^{HA}+\left[ H^{+} \right] \right)^{2}})$ | (S33) |
| --- | --- |

For each buffer a system of differential algebraic equations resulted: Equations (S12), (S29) and the Mooney model surface pH equation for phosphate and equations (S12), (S33) and the RNE model surface pH equation for bicarbonate.

For the case of non-equal diffusivity between the ionized and non-ionized drug species (e.g. to investigate the possible effect of near complete free acid dimerization), equation (S1) needs to be adjusted by splitting the drug flux into ionized and non-ionized species’ fluxes as follows:

| $\frac{dq_{diss}}{dt}=\sum_{i=1}^{n=6} (\frac{3 f_{i} Dose}{h_{i} {r_{ini}}_{i}\rho}\left( 1-\frac{{q_{diss}}_{i}}{f_{i} Dose} \right)^{\frac{2}{3}} \left( D_{HA}(is-{[HA]}_{bulk})+D_{A}(is\times\frac{K_{a}^{HA}}{\left[ H^{+} \right]_{s}}-{[A^{-}]}_{bulk}) \right))$ | (S34) |
| --- | --- |

| ${[HA]}_{bulk}=\frac{{[H^{+}]}_{bulk}\sum_{i=1}^{n=6} {q_{diss}}_{i}}{V_{diss}(K_{a}^{HA}+{[H^{+}]}_{bulk})}$ | (S35) |
| --- | --- |

| ${[A^{-}]}_{bulk}= \frac{K_{a}^{HA}\sum_{i=1}^{n=6} {q_{diss}}_{i}}{V_{diss}(K_{a}^{HA}+{[H^{+}]}_{bulk})}$ | (S36) |
| --- | --- |

where ${[H^{+}]}_{bulk}$ is the bulk proton molarity represented by $\left[ H^{+} \right]$ in equations (S17)-(S33). D_HA_ and D_A_ represent the diffusion coefficients of the protonated and deprotonated species respectively. In this case it was assumed that almost all the protonated ibuprofen molecules existed in a dimerized state to test the sensitivity of the predictions to dimerization.

The diffusion coefficient of the dimer can be estimated by using an equation developed by Avdeef: [3]

| ${log D}_{j}=-4.131-0.4531 log {MW}_{j}$ | (S37) |
| --- | --- |

Where D_j_ and MW_j_ are the diffusion coefficient and the molecular weight of the jth species respectively. Since this empirical fit was performed for diffusivity data normalized to 25 °C, the resulting value was multiplied by 1.339 to give the diffusivity at 37 °C. [3]

The required physicochemical parameters for ibuprofen, phosphate and bicarbonate are outlined in **Table S1**. The values pertain to a temperature of 37 °C and an ionic strength of 0.15 M.

**Table S1** Physicochemical parameters used in simulations

| **Material** | **Parameter** | **Value** | **Reference** |
| --- | --- | --- | --- |
| Ibuprofen | pKa | 4.41 | [4] |
|  | Intrinsic solubility | 2.8x10^-4^ M | [4] |
|  | Diffusion coefficient (both protonated and deprotonated) | 7.93x10^-6^ cm^2^ s^-1^ | [4] |
|  | True density | 1.12 g cm^-3^ | [5] |
| Phosphate buffer | pKa | 6.75 | [6]^a^ [7]^b^ |
|  | Diffusion coefficient of H_2_PO_4_^-^ | 11.5x10^-6^ cm^2^ s^-1^ | [8] |
|  | Diffusion coefficient of HPO_4_^2-^ | 11.5x10^-6^ cm^2^ s^-1^ | [8] |
| Bicarbonate buffer | pKa of H_2_CO_3_ | 3.3 ^c^ | [4] |
|  | Hydration rate constant for CO_2_ | 75.5 s^-1^ | [4] |
|  | Dehydration rate constant for H_2_CO_3_ | 0.109 s^-1^ | [4] |
|  | Diffusion coefficient of CO_2_ | 24.9x10^-6^ cm^2^ s^-1^ | [4] |
|  | Diffusion coefficient of H_2_CO_3_ | 18.08x10^-6^ cm^2^ s^-1^ | [4] |
|  | Diffusion coefficient of HCO_3_^-^ | 14.6x10^-6^ cm^2^ s^-1^ | [4] |
| Water | Diffusion coefficient of H^+^ | 100x10^-6^ cm^2^ s^-1^ | [9] |
|  | Diffusion coefficient of OH^-^ | 63x10^-6^ cm^2^ s^-1^ | [9] |
|  | K_w_ | 4.18x10^-14^ M^2 d^ | [10] |
|  | Kinematic viscosity | 0.00696 cm^2^ s^-1^ | [2] |
| ^a^ Temperature effect calculated based on this source  ^b^ Ionic strength effect calculated based on this source  ^c^ Only H_2_CO_3_ deprotonation without the preceding hydration of CO_2_  ^d^ Calculated using the equation of Sweeton et al. | | | |

References

1. Wang J, Flanagan DR. General solution for diffusion-controlled dissolution of spherical particles. 1. Theory. J Pharm Sci. 1999;88:731–8. doi:10.1021/js980236p.

2. Avdeef A, Tsinman K, Tsinman O, Sun N, Voloboy D. Miniaturization of powder dissolution measurement and estimation of particle size. Chem Biodivers. 2009;6:1796–811. doi:10.1002/cbdv.200900082.

3. Avdeef A. Leakiness and size exclusion of paracellular channels in cultured epithelial cell monolayers-interlaboratory comparison. Pharm Res. 2010;27:480–9. doi:10.1007/s11095-009-0036-7.

4. Al-Gousous J, Salehi N, Amidon GE, Ziff RM, Langguth P, Amidon GL. Mass Transport Analysis of Bicarbonate Buffer: Effect of the CO2-H2CO3 Hydration-Dehydration Kinetics in the Fluid Boundary Layer and the Apparent Effective p Ka Controlling Dissolution of Acids and Bases. Mol Pharm. 2019;16:2626–35. doi:10.1021/acs.molpharmaceut.9b00187.

5. D H, FC C. A Modern Approach to the Heckel Equation: The Effect of Compaction Pressure on the Yield Pressure of Ibuprofen and its Sodium Salt. J Nanomed Nanotechnol 2016. doi:10.4172/2157-7439.1000381.

6. Samuelsen L, Holm R, Lathuile A, Schönbeck C. Buffer solutions in drug formulation and processing: How pKa values depend on temperature, pressure and ionic strength. International Journal of Pharmaceutics. 2019;560:357–64. doi:10.1016/j.ijpharm.2019.02.019.

7. Manov GG, Bates RG, Hamer WJ, Acree SF. Values of the Constants in the Debye—Hückel Equation for Activity Coefficients 1. J. Am. Chem. Soc. 1943;65:1765–7. doi:10.1021/ja01249a028.

8. Sheng JJ, McNamara DP, Amidon GL. Toward an in vivo dissolution methodology: a comparison of phosphate and bicarbonate buffers. Mol Pharm. 2009;6:29–39. doi:10.1021/mp800148u.

9. Uekusa T, Avdeef A, Sugano K. Is equilibrium slurry pH a good surrogate for solid surface pH during drug dissolution? Eur J Pharm Sci. 2022;168:106037. doi:10.1016/j.ejps.2021.106037.

10. Sweeton FH, Mesmer RE, Baes CF. Acidity measurements at elevated temperatures. VII. Dissociation of water. J Solution Chem. 1974;3:191–214. doi:10.1007/BF00645633.
